# Does smooth endoplasmic reticulum aggregation in oocytes impact the chromosome aneuploidy of the subsequent embryos? A propensity score matching study

**DOI:** 10.1186/s13048-023-01135-z

**Published:** 2023-03-24

**Authors:** Meng Wang, Limin Gao, Qiyu Yang, Rui Long, Yini Zhang, Lei Jin, Lixia Zhu

**Affiliations:** Reproductive Medicine Center, Tongji Hospital, Tongji Medical College, Huazhong University of Science and Technology, Wuhan, 430030 China

**Keywords:** Smooth endoplasmic reticulum aggregation, Chromosome aneuploidy, Clinical outcome, Oocyte dysmorphism, Preimplantation genetic testing

## Abstract

**Background:**

The appearance of smooth endoplasmic reticulum aggregation (SERa) is one of the most common dysmorphic phenotypes of oocytes, however, the impact of SERa occurrence on in vitro fertilization (IVF) outcomes is controversial. This study aimed to investigate the impact of SERa in oocytes on the aneuploidy of the subsequent embryos in IVF.

**Methods:**

In this retrospective cohort study, a total of 114 intracytoplasmic sperm injection (ICSI) cycles with the appearance of SERa undergoing preimplantation genetic testing for aneuploidy (PGT-A) were enrolled, and among them there were 323 SERa(+) oocytes and 1253 sibling unaffected oocytes. The 907 PGT-A cycles without SERa during the same period were enrolled as controls. A propensity score matching of 1:1 ratio between these two groups resulted in 113 matched cycles. The outcome parameters between the SERa(+) cycles/oocytes and the controls were compared. IVF laboratory outcomes, PGT-A outcomes, and clinical and neonatal outcomes were the main outcomes.

**Results:**

Increased abnormal fertilization rate and reduced blastocyst formation rate can be observed in both SERa(+) cycles and oocytes, some other parameters on developmental potential, such as available embryo rate at Day 3 and available blastocyst rate, were also impaired in the case of SERa occurrences. Among the 910 blastocysts for PGT-A, the percentage of euploid embryos was similar between the matched cohorts, while an unpredicted increase of the proportions of euploid in the SERa(+) oocytes, compared to the SERa(-) oocytes. Moreover, there was no significance in terms of clinical and neonatal outcomes, such as implantation rate, biochemical pregnancy rate, clinical pregnancy rate, miscarriage rate, and live birth rate, regardless of the presence of SERa in cycles and oocytes.

**Conclusions:**

The appearance of SERa within mature oocytes has no significant impact on the aneuploidy of subsequent blastocysts. It is recommended to utilize these oocytes, especially for those with few oocytes or advanced maternal age, which is likely to increase the cumulative pregnancy rate. This study may offer evidence to assist embryologists to make clinical decisions concerning SERa(+) oocytes more consciously and rationally.

**Supplementary Information:**

The online version contains supplementary material available at 10.1186/s13048-023-01135-z.

## Background

It has been shown that the smooth endoplasmic reticulum (SER) regulates early embryonic development through energy accumulation and plays a key role in calcium storage and redistribution, which is significant for the process of oocyte activation and fertilization [1]. Smooth endoplasmic reticulum aggregation (SERa), one of the most common cytoplasmic dysmorphisms in oocytes, appears as multiple dispersed spherical aggregates surrounded by mitochondria [2, 3]. In the process of in vitro fertilization (IVF), the prevalence of SERa occurrences ranges from 4 to 23% in stimulation cycles and the proportion of SERa(+) oocytes was approximately 17.6 to 29.1% per affected cycle [4]. The underlying mechanism responsible for the presence of SERa in oocytes remains unknown yet, while some studies demonstrated that it was related to the utilization of exogenous gonadotropin in controlled ovarian hyperstimulation (COH) [5].

The impact of the occurrence of SERa in oocytes on IVF outcomes is controversial. Some studies reported the fertilization rate, pregnancy rate, and malformation rate in neonates were not different between SERa(+) and SERa(-) oocytes [6, 7]. While some other studies suggested a lower implantation rate in SERa(+) cycles [8], as well as a lower clinical pregnancy rate [9]. Moreover, it has been shown that the presence of SERa may increase the risk of birth defects [5]. Considering the existing conflicting data regarding the association between SERa(+) oocytes and IVF outcomes, policies towards the use of SERa(+) oocytes are not homogeneous, and about 14% of the IVF centers discarded these oocytes in a multicentre survey study [10]. The revised Alpha/ESHRE consensus in 2017 recommended a case-by-case approach for SERa(+) oocytes [11]. However, this approach may not be constructive enough for embryologists to manage SERa(+) oocytes, especially when the number of available oocytes is limited in some cases [4]. A normal chromosome number is essential for embryonic development, and preimplantation genetic testing for aneuploidy (PGT-A) is effective for the selection of euploid embryos [12]. Thus, chromosome aneuploid analyses are likely to assist embryologists to select euploid embryos originating from SERa(+) oocytes. While currently, no previous studies have ever explored the relationship between SERa(+) oocytes and the number of chromosomes in the subsequent embryos.

The present study aimed to investigate the impact of SERa occurrences on chromosome aneuploidy of the subsequent embryos as well as oocyte developmental competency and clinical outcomes in IVF. This study may offer evidence to assist embryologists to make clinical decisions concerning SERa(+) oocytes more consciously and rationally.

## Methods

### Study design and population

It was a single-center retrospective cohort study performed in Tongji Hospital, Tongji Medical College, Huazhong University of Science and Technology. Infertility couples with identified SERa in oocytes and aneuploid analysis in embryos by PGT-A in the IVF center between November 2016 and October 2021 were included in this study, which was approved by the Ethical Committee of Tongji Hospital (TJ-IRB20211280). Signed informed consent and information use forms were obtained from patients. The exclusion criteria were as follows: (a) number of available oocytes less than 3; (b) total fertilization failure; (c) other identified types of morphological abnormality in oocytes; (d) identified genetic mutations correlated to gametes and embryo development; (e) lost to follow-up and important information missed. SERa(+) oocytes were referred to as those oocytes which had one or more visible SERa after denudation, and the SERa(+) cycles had at least one SERa(+) oocyte in the same oocyte cohort. The oocytes in the SERa(+) cycles groups were further divided into the SERa(+) oocytes group and the sibling SERa(-) oocytes groups, in which the oocytes were morphologically unaffected (Fig. S1). The patients who underwent PGT-A with normal oocyte morphology without SERa during the same period were enrolled as the control group. To eliminate the imbalance of the number and distribution of samples between the SERa(-) cycles group and the SERa(+) cycles group, a propensity score matching of a 1:1 ratio was performed.

### Oocyte retrieval, fertilization and embryo culture

The details of COH protocols were previously well-described [13]. Briefly, it included the gonadotropin-releasing hormone (GnRH) agonist protocol, the GnRH antagonist protocol, and other protocols such as the mild stimulation and luteal phase stimulation protocols. The follicular growth was monitored by transvaginal ultrasound. When there were two to three dominant follicles with a diameter over 18 mm, recombinant human chorionic gonadotropin (HCG, Livzon, China) was administered for the trigger. Transvaginal ultrasound-guided oocyte retrieval was performed 36–38 h later. Cumulus-oocyte complexes (COCs) were collected and cultured in incubators at 37℃, 6% CO_2_.

Followed by mechanical pipetting in G-MOPS plus medium (Vitrolife, Gothenburg, Sweden) for denudation, cumulus-oocyte complexes (COCs) were exposed to 80 U/L hyaluronidase (Irvine Scientific, the United States), and denuded oocytes were further cultured in G1-plus medium (Vitrolife, Gothenburg, Sweden) for another 1–2 h before spermatozoon injection [14]. Generally, pronuclei (PN) assessments were performed 16–18 h after fertilization [15]. Fertilization was confirmed by the presence of 2PN and the extrusion of the second polar body. The time-lapse system (Vitrolife, Denmark) was used to monitor and record the processes of embryo development. Zygotes were cultured in sequential media (G1-plus and G2-plus media, Vitrolife, Gothenburg, Sweden) to the blastocyst stage (D5/D6).

### Morphological assessment of oocytes and embryos

Oocyte morphology and maturity were evaluated after degranulation under the inverted microscope and re-evaluated while ICSI operation. SERa(+) oocytes referred to those mature oocytes with the aggregations exhibiting round flat disks corresponding to large clusters of tubular SER in the ooplasm. SERa was ascertained by two senior embryologists when spermatozoon injection and marked detailed. Normally fertilized oocytes were cultured. The morphology of embryos at the cleavage stage was assessed based on the number and variation of blastomeres as well as fragments as previously described [16]. Blastocysts were scored morphologically based on the Gardner scoring criteria [17].

### Chromosome aneuploid analyses and transfer strategy

Three to eight trophectoderm cells of blastocysts with a morphological score of 3BC and above at D5 or D6 were collected for biopsy. Followed by next-generation sequencing (NGS), multiple annealing- and looping-based amplification cycles (MALBAC) was utilized for amplification according to the manufacturer’s recommendations. The details have been well described in previous studies [18]. The results of PGT-A were as follows: “Euploid” referred to an euploid embryo available for transfer. “Aneuploid” demonstrated chromosomal copy number unconformable to euploidy. “Mosaic” indicated that the embryos have two or more cell populations with a different chromosomal set, which were generally ineligible for transfer. “N/A” was defined as amplification failure. Single embryo transfer was performed based on the results of PGT-A analyses and embryo assessment in our IVF center in the frozen-thawed embryo transfer (FET) cycle. The surplus embryos with normal chromosomal sets were cryopreserved.

### Outcome assessments

The outcomes in the current study were mainly IVF laboratory outcomes, PGT-A outcomes, and clinical outcomes. For IVF laboratory outcomes, the normal fertilization rate and available blastocyst rate were the primary outcomes. The secondary outcomes included the mature oocyte, the abnormal fertilization rate, the cleavage rate, the available embryo rate at D3, and the blastocyst formation rate. The details of computing formulae were well described previously [19]. Blastocyst formation rate referred to the number of blastocysts formed divided by the number of embryo continually cultured on Day 3. Available blastocysts referred to blastocysts scored as 3BC or above according to the Gardner scoring system. The available blastocyst rate was the number of available blastocysts divided by the number of embryos continually cultured on Day 3. For PGT-A outcomes, it can be classified into “euploid”, “aueuploid” and “mosaic” as mentioned above. For clinical outcomes, the primary outcome was the live birth rate, other parameters including the implantation rate, the biochemical pregnancy rate, the clinical pregnancy rate, and the miscarriage rate were the secondary outcomes. The assessments of clinical and biochemical pregnancy were based on the standard of our IVF center [19]. Briefly, a rise in serum HCG level was regarded as biochemical pregnancy, and clinical pregnancy was defined as ultrasonographic visualization of fetal heart activities in gestational sacs 5 weeks after embryo transfer.

### Statistical analyses

Data were analyzed using Statistical Package for Social Sciences software (SPSS, version 26.0, IBM, the United States). At first, the Shapiro–Wilk normality test was performed in the continuous variables, and non-normal distributed variables were presented as medians (first quartile, third quartile), the comparisons of which were accomplished using Mann–Whitney *U* test. The categorical variables were exhibited as % (n), and the differences were analyzed using the chi-squared test or Fisher’s exact test as appropriate.

A propensity score matching was performed, and the following baseline characteristics were matched: age (years), body mass index (BMI, kg/m^2^), follicle-stimulating hormone (FSH), anti-Müllerian hormone (AMH), the basal antral follicle count (AFC), ART attempts, infertility duration (years), infertility type (primary and secondary) and COH protocols (GnRH agonist protocol, GnRH antagonist protocol, and other protocols). The matching algorithm was nearest neighbor random matching without replacement, and the match ratio was 1:1 with a tolerance of 0.1. A *P-*value < 0.05 in two-tailed hypothesis tests was considered to be of statistical significance.

## Results

**Fig. 1:**
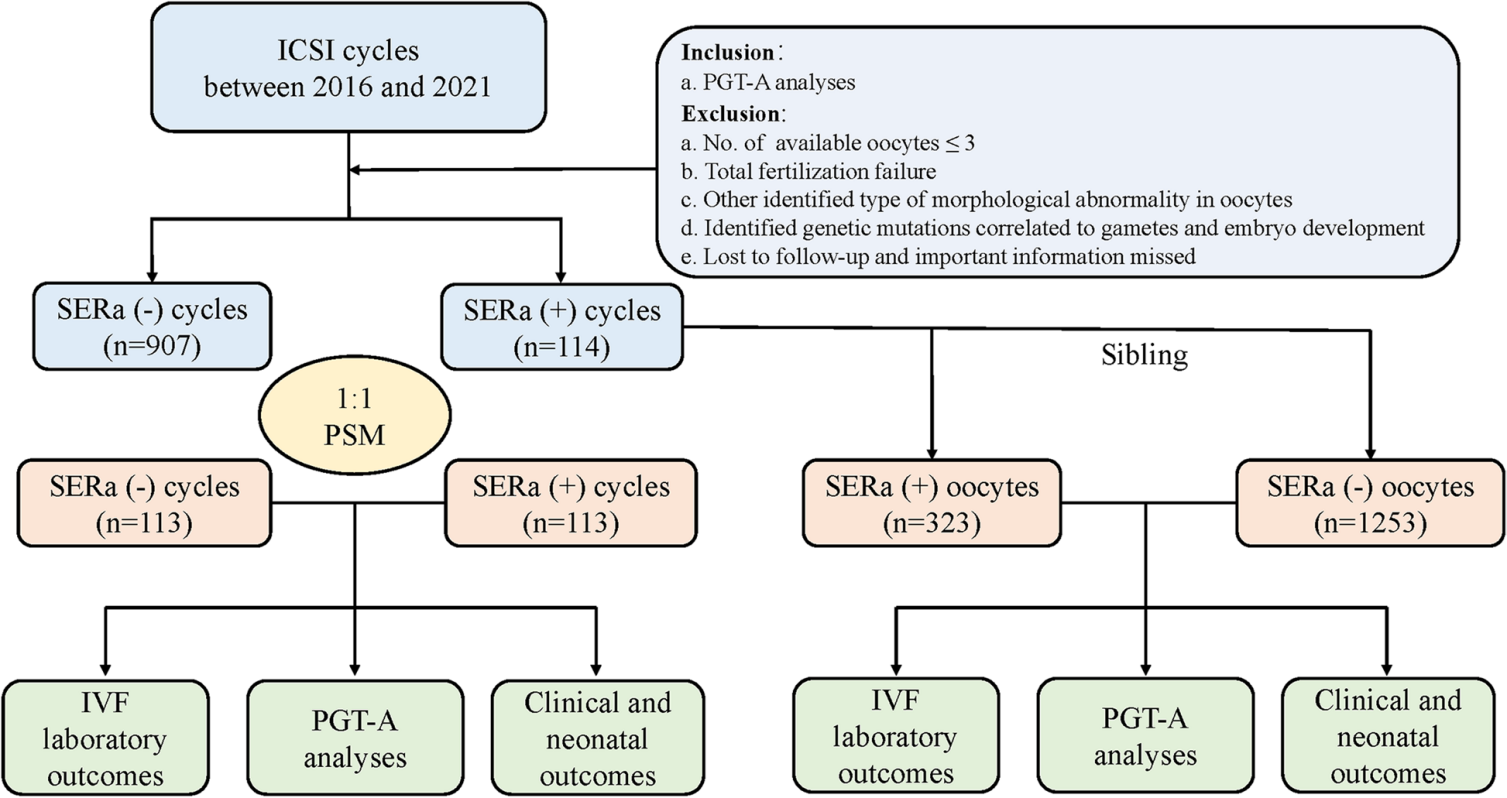
Flow chart of the study. Note: *SERa* Smooth endoplasmic reticulum aggregation, *IVF* In vitro fertilization, *PSM* Propensity score matching, *PGT-A* Preimplantation genetic testing for aneuploidy

A total of 114 PGT-A cycles with the presence of SERa(+) oocytes (*n* = 114) were enrolled as the case group in this study, of which there were 323 SERa(+) oocytes and sibling 1253 SERa(-) oocytes (Fig. 1)**.** Then 907 PGT-A cycles without SERa during the same period were enrolled as controls. To eliminate the imbalance of characteristics, a propensity score matching of 1:1 ratio was performed to create a comparable matched cohort and resulted in 113 matched cycles. No significant difference was shown between the groups regarding the baseline characteristics after matching (Table 1). In the SERa(+) cycles group,. Moreover, after matching, there were no significant differences in terms of the parameters of ovarian responses, including gonadotropin dosage, gonadotropin duration, estradiol level on HCG day, progesterone level on HCG day, endometrium thickness on HCG day, and the number of large follicles (Table 1).

**Table 1:** Baseline characteristics after matching

	Case (*n* = 113) SERa(+) cycle	Control (*n* = 113) SERa(-) cycle	*P* value
Age (years)	30 (28, 34)	30 (28, 33)	0.807
BMI (kg/m^2^)	21.5 (20.3, 23.5)	21.6 (19.5, 23.4)	0.353
FSH (IU/L)	6.7 (6.1, 8.4)	6.8 (5.9, 8.0)	0.506
AMH (ng/mL)	4.0 (2.5, 7.5)	4.3 (2.7, 7.5)	0.583
AFC	14 (9, 22)	15 (11, 20)	0.479
ART attempts	1 (1, 1)	1 (1, 1)	0.277
Infertility durations (years)	2.0 (0.0, 3.0)	1.5 (0.7, 3.0)	0.984
Infertility types			0.786
Primary, %(n)	40.7 (46)	38.9 (44)	
Secondary, %(n)	59.3 (67)	61.1 (69)	
COH protocols			0.905
GnRH agonist protocol, %(n)	62.8 (71)	65.5 (74)	
GnRH antagonist protocol, %(n)	30.1 (34)	27.4 (31)	
Other protocol^a^, %(n)	7.1 (8)	7.1 (8)	
Gonadotropin dosage (IU)	2475 (1823, 3000)	2325 (1860, 2963)	0.946
Gonadotropin duration (days)	10 (9, 11)	10 (9, 12)	0.381
Estradiol on HCG day (pg/mL)	3000 (1756, 4386)	2417 (1635, 4435)	0.339
Progesterone on HCG day (ng/mL)	0.9 (0.6, 1.3)	0.9 (0.6, 1.3)	0.746
Endometrium thickness on HCG day (mm)	11.3 (9.0, 13.3)	10.6 (9.1, 12.7)	0.510
No. of large follicles	12 (9, 16)	12 (8, 16)	0.574

*BMI* Body mass index, *FSH* Follicle stimulation hormone, *AMH* Anti-müllerian hormone, *AFC* Antral follicle counting, *ART* Assisted reproductive technology, *COH* Controlled ovarian hyperstimulation, *GnRH* Gonadotrophin releasing hormone, *HCG* Human chorionic gonadotropin

^a^Other protocol: including mild stimulation and luteal phase stimulation protocols

Continuous variables were presented as median (first quartile, third quartile)

Categorical variables were presented as % (n)

*P* < 0.05 was considered statistically significant

The IVF laboratory outcomes were presented in Table 2. In the SERa(+) cycles group, 1875 oocytes were retrieved and 1718 oocytes were retrieved in the SERa(-) cycles group after matching. Moreover, there were 1536 mature oocytes in the 113 SERa(+) cycles after matching, and 21.9% (337/1536) were SERa(+) oocytes. Compared to the SERa(-) cycles group, increased abnormal fertilization rate (*P* = 0.029), lower cleavage rate (*P* < 0.001), decreased blastocyst formation rate (*P* = 0.041), and reduced available blastocyst rate (*P* = 0.015) can be observed in the SERa(+) cycles group, indicating the restricted developmental potential of oocytes in the SERa(+) cycles group. Similar results were also shown in the comparison of the SERa(+) oocytes group and the sibling SERa(-) oocytes group. The SERa(+) oocytes had a much higher abnormal fertilization rate (*P* < 0.001), lower available embryo rate at D3 (*P* < 0.001), and reduced blastocyst formation rate (*P* = 0.021), despite increased available blastocyst rate (*P* = 0.001).

**Table 2:** Embryo developmental outcomes of SERa + cycles/oocytes

	SERa(+) cycle	SERa(-) cycle	*P* value	SERa(+) oocyte	SERa(-) oocyte	*P* value
Cycles	113	113				
No. of oocytes retrieved	1875	1718				
No. of mature oocytes	1536	1390		323	1253	
Mature oocyte rate, %(n)	81.9 (1536)	80.9 (1390)	0.436			
Normal fertilization rate, %(n)	73.3 (1126)	73.7 (1024)	0.825	71.2 (230)	73.5 (921)	0.407
Abnormal fertilization rate, %(n)	1.7 (26)	0.8 (11)	0.029	4.3 (14)	1.0 (13)	< 0.001
Cleavage rate, %(n)	95.0 (1094)	98.6 (1021)	< 0.001	95.9 (234)	94.6 (884)	0.427
Available embryo rate at D3, %(n)	91.4 (1053)	92.8 (960)	0.245	83.2 (203)	93.4 (872)	< 0.001
Blastocyst formation rate, %(n)	66.3 (698)	70.5 (677)	0.041	59.6 (121)	68.1 (597)	0.021
Available blastocyst rate, %(n)	44.2 (465)	49.6 (476)	0.015	53.7 (110)	41.9 (363)	0.001

Categorical variable was presented as % (n)

*P* < 0.05 was considered statistically significant

As shown in Table 3, 941 available blastocysts from 226 cycles after matching were obtained. Due to the request of patients, PGT-A was not carried out in 31 of them, and the rest (96.7%, 910/941) were assessed for PGT-A. Except for the cases of amplification failure, 890 (97.8%) of them were with outcomes of chromosomal sets. There were no significant differences regarding the percentages of embryos with different numbers of chromosomes between the SERa(+) cycles group and the SERa(-) cycles group. Furthermore, in the 114 SERa(+) cycles before matching, there were 102 blastocysts originating from SERa(+) oocytes and 358 blastocysts originating from sibling SERa(-)oocytes, and the remaining 447 embryos were with outcomes of chromosomal sets. Surprisingly, there were more euploid embryos in the SERa(+) oocytes group than the sibling SERa(-) oocytes (50.0% *vs* 37.6%, *P* = 0.028).

**Table 3:** Chromosome aneuploidy analyses outcomes of SERa + cycles/oocytes

	SERa(+) cycle	SERa(-) cycle	*P* value	SERa(+) oocyte	SERa(-) oocyte	*P* value
No. of blastocysts for PGT-A tests	452	458		102	358	
No. of blastocysts with chromosomal set outcomes	439	451		96	351	
Euploid, %(n)	40.1 (176)	40.1 (181)	0.990	50.0 (48)	37.6 (132)	0.028
Aneuploid, %(n)	46.0 (202)	46.1 (208)	0.975	37.5 (36)	48.4 (170)	0.057
Mosaic, %(n)	13.9 (61)	13.7 (62)	0.949	12.5 (12)	14.0 (49)	0.738

*PGT-A* Preimplantation genetic testing for aneuploidy

Categorical variable was presented as % (n)

*P* < 0.05 was considered statistically significant

In our study, single-embryo-transfer strategy was performed. In the matched groups, 155 euploid blastocysts were transferred (73 in the SER(+) cycles group and 82 in the SER(-) cycles group), and among the 73 embryos in the SER(+) cycles, 22 of them were originated from SERa(+) oocytes. There were no significant differences between the two groups in terms of implantation rate, biochemical pregnancy rate, clinical pregnancy rate and miscarriage rate (Table 4). Similarly, 22 embryos were transferred in the SERa(+) oocytes group and 51 in the sibling SERa(-) oocytes group. No significant differences were observed between the groups regarding the mentioned clinical outcomes parameters. Moreover, in the SERa(+) and SERa(-) cycles groups, 47 and 44 women delivered, respectively, with 9 and 8 ongoing pregnancies by the end of this study in the corresponding groups, respectively. In SERa(+) and SERa(-) oocytes groups, live births were confirmed in 11 and 36 women, respectively, with 6 and 3 ongoing pregnancies. In the SERa(+) oocytes group, one baby derived from SERa(+) oocytes was detected with patent foramen ovale 6 months after birth, and no birth defects or complications were found in the rest of the newborns.

**Table 4:** Clinical outcomes of SERa + cycles/oocytes

	SERa(+) cycles	SERa(-) cycles	*P* value	SERa(+) oocyte	SERa(-) oocyte	*P* value
No. of embryos transferred	73	82		22	51	
Implantation rate, %(n)	83.6 (61)	75.6 (62)	0.222	83.3 (18)	86.4 (43)	0.733
Biochemical pregnancy rate, %(n)	89.0 (65)	85.4 (70)	0.496	86.4 (19)	90.2 (46)	0.691
Clinical pregnancy rate, %(n)	83.6 (61)	75.6 (62)	0.222	83.3 (18)	86.4 (43)	0.733
Miscarriage rate, %(n)	8.2 (5)	16.1 (10)	0.179	5.0 (1)	10.5 (4)	0.650

Single-embryo-transfer strategy was performed

Categorical variable was presented as % (n)

*P* < 0.05 was considered statistically significant

## Discussion

In this retrospective cohort study, we investigated the impact of the appearance of SERa in oocytes on chromosome aneuploidy of the subsequent blastocysts as well as embryo developmental competency and clinical outcomes in IVF. It was found that embryonic developmental potential in SERa( +) oocytes and SERa(+) cycles were partly compromised, whereas the results of chromosome aneuploidy and clinical outcomes were similar between the cohorts.

The appearance of SERa is one of the most common dysmorphic phenotypes of oocytes [20], while how SERa occurs in oocytes remains unclear and controversial. Our data showed that the prevalence of SERa was 11.1% in IVF cycles, which were considered reasonable and in accordance with previously reported [21]. It was found that the formation of SERa was related to ovarian stimulation protocols, especially the doses and duration of gonadotropin administration [6, 9]. It has been reported that a high dose of exogenous gonadotropin impairs oocyte regulatory mechanism, resulting in oocyte cytoplasmic dysmorphism and oocyte mature disorder, chromosome disarrangement, and subsequently affecting the developmental potential of oocytes and embryos [22]. Another study found the absence of SERa in oocytes from unstimulated women, suggesting the strong relationship between the presence of SERa and the stimulation with extracorporeal gonadotropins [23]. Similarly, it was also previously shown that the occurrence of SERa was positively related to estradiol level on HCG day as a consequence of ovarian hyperstimulation [4]. In our study, the choice of COH protocol, the dosage and duration of gonadotropin administration were similar between the groups before and after matching, revealing that the etiology of such a cytoplasmic dysmorphism of oocytes is ambiguous. Meanwhile, SERa is speculated to originate from genetic abnormalities, since the reoccurrence of SERa can be observed in different cycles of the same patient [5, 24]. This phenomenon was also observed in some patients in our cohort. Although the mechanism underlying the origin of SERa in oocytes was unknown, the potential predictive factors of SERa occurrence should be further explored.

As the organelle for the storage and release of calcium, the endoplasmic reticulum is significant and necessary in the process of oocyte activation, fertilization, and energy accumulation [25–27]. Thus the abnormal aggregates of SER in oocytes might greatly impair oocyte physiology via the aberrant calcium signals, resulting in decreased fertilization rate and reduced embryo quality, which was reinforced by a large number of studies [28, 29]. In this study, we compared the embryological outcomes in matched SERa(+) and SERa(-) cycles as well SERa(+) and sibling SERa(-) oocytes. Increased abnormal fertilization rate and decreased blastocyst formation rate were observed regardless of cycles and oocytes exhibiting SERa. Besides, the cleavage rate in SERa(+) cycles and available embryo rate at D3 in SERa(+) oocytes were reduced, which was consistent with previous studies [4]. Moreover, another study also found that the presence of SERa in oocytes can negatively affect blastocyst quality and the speed of blastocyst development [30]. Interestingly, although the available blastocyst rate was much lower in SERa(+) cycles than that in SERa(-) cycles, this value dramatically increased in the SERa(+) oocyte compared to controls. A recent study analyzed the transcriptome of SERa(+) and SERa(-) oocytes, and it was found that genes involved in the process of mitosis and meiosis, the organization of cytoskeleton and microtubules, and the structure and activity of the mitochondria were down-regulated in SERa(+) oocytes, while genes related to the process of cell proliferation, differentiation and embryogenesis were up-regulated [31], which partly explained the increased available blastocyst rate in SERa(+) oocytes in our study.

Although the developmental parameters of some embryos derived from SERa(+) oocytes were relatively normal, they cannot be treated as a “normal” one by embryologists due to various concerns about safety. It was shown that oocytes exhibiting severe cytoplasmic dysmorphism were with a higher incidence of aneuploidy and chromosomal scattering [32], however, currently, no available study aimed to investigate the potential effects of the presence of SERa in oocytes on aneuploidy risk in embryos. In our study, we included 910 cycles with available blastocysts for PGT-A, and it was found that the percentage of euploid embryos was similar between the groups. Surprisingly, it was suggested that the SERa(+) oocytes had a higher proportion of euploid embryos compared to the sibling SERa(-) oocytes. Some reasons may elucidate these phenomena. On one hand, SERa(+) oocytes are normally companied with dysplasia, fertilization failure, polyspermy, and poor embryonic development potential, which lead to the early phase elimination of embryos originating from SERa(+) oocytes. Those SERa(+) oocytes, which are able to develop into the blastocyst stage, have already undergone the process of self-selection, resulting in an increased capacity for self-repair and renewal. Meanwhile, considering increasing evidence for embryo self-correction of chromosomal abnormalities, it was likely to be associated with the up-regulation of genes that participated in cell self-checking, repair, and self-correction during the process of cell proliferation and differentiation [33, 34]. It is urgent to clarify the expression of relevant genes in blastocysts to verify these deductions. Meanwhile, the uneven number of cases within the groups might also result in the differences in euploidy rate. In the future, mechanism explorations and clinical studies with a larger samples size are needed to explore the underlying mechanism. Based on the above results, we still recommend that SERa(+) oocytes are not supposed to be discarded, especially when there are no available sibling SERa(-) oocytes. Extended culture and blastocyst transfer will help the embryologist screen embryos with better developmental potential and normal chromosomal sets when dealing with SERa(+) oocytes and embryo selection.

With regard to clinical outcomes, the conclusions of previous studies were discordant. In most recent studies, the clinical pregnancy rate between the SERa (+) and SERa(-) cycles was similar [4, 35], and so was our study, while a decreased clinical pregnancy rate in SERa (+) cycles was also reported in another study [9]. As for SERa(+) oocytes, it was suggested that the presence of SERa(+) in oocytes was relevant to poorer clinical pregnancy outcomes. Only one study reported a higher miscarriage rate in SERa-affected cycles [5], whereas most studies regarding miscarriage rate including ours suggested that no significant difference was exhibited in terms of this parameter [4]. Although the data about birth defects in our study was limited, previous studies have already reported malformations or genetic abnormalities in newborns originating from SER(+) cycles or oocytes, such as Beckwith-Wiedmann syndrome, diaphragmatic hernia, multiple malformations, and cardiovascular defects [5, 9, 24]. However, several publications have suggested that healthy infants can derive from SERa(+) oocytes [6, 36, 37]. In this context of controversy, larger studies and longer follow-ups focused on the impact of SERa on clinical and neonatal outcomes are mandatory and urgent.

The strategy to deal with SERa(+) oocytes varied with time. The Istanbul Consensus in 2011 advised the abandonment of SERa(+) oocytes due to a higher risk of malformations reported in neonates originating from these oocytes [38]. While in 2017, a case-by-case approach was recommended by the revised Alpha/ESHRE consensus, and a strict follow-up was also advised [11]. However, a case-by-case approach may be confusing for embryologists to make decisions in the management of SERa(+) oocytes. As the first study to investigate the relationship between the occurrence of SERa and the number of chromosomes, our results suggested that although the laboratory outcomes of SERa(+) oocytes might not be as good as the sibling unaffected oocytes, the number of chromosomes of the blastocysts originated from oocytes with such a dysmorphism were not likely to be affected. Therefore, based on our results, it was encouraged to utilize SERa(+) oocytes for fertilization and even embryo transfer. It was of great significance for infertility patients, especially for those with few oocytes or advanced age females, which is likely to increase the cumulative pregnancy rate.

To the best of our knowledge, this is the first report to investigate the impact of SERa oocytes on chromosome aneuploidy of subsequent blastocysts. We found a similar proportion of euploidy blastocysts regardless of the presence of SERa in oocytes. However, there were still several limitations. At first, it was a single-center retrospective cohort study, and the sample size was limited. Investigations with a larger sample size among multiple centers are needed to reinforce the results. Moreover, this study only enrolled IVF patients with aneuploid analysis, which may cause selection bias in the population. In addition, the main outcome was the number of chromosomes, and the impact of SERa occurrence on the genetics or epigenetics of the embryos needs further exploration. Furthermore, the number of neonates derived from SERa(+) oocytes was limited in our study, and a long-term follow-up of children is required. Additionally, PGT-A tests were performed in embryos at the blastocyst stage in our study, and whether the embryo aneuploidies originated from oocytes or spermatozoa remains unclear and needs further investigation.

## Conclusions

In conclusion, the presence of SERa has no significant impact on the chromosome aneuploidy of the subsequently developed blastocysts. Although the developmental parameters of the SERa(+) oocytes were partly impaired compared to the sibling unaffected oocytes, it is recommended to utilize these oocytes for fertilization, especially for those with few oocytes or advanced maternal age females.

## Supplementary Information

**Additional file 1: Figure S1. **Image of SERa oocyte under time-lapse monitoring system.

## Data Availability

The datasets used and/or analyzed during the current study are available from the corresponding author on reasonable request.

## Abbreviations

AFC: Antral follicle count
AMH: Anti-Müllerian hormone
BMI: Body mass index
COCs: Cumulus-oocyte complexes
COH: Controlled ovarian hyperstimulation
FET: Frozen-thawed embryo transfer
FSH: Follicle-stimulating hormone
GnRH: Gonadotropin-releasing hormone
HCG: Human chorionic gonadotropin
ICSI: Intracytoplasmic sperm injection
IVF: in vitro Fertilization
MALBAC: Multiple annealing- and looping-based amplification cycles
NGS: Next-generation sequencing
PGT-A: Preimplantation genetic testing for aneuploidy
PN: Pronucleus
SER: Smooth endoplasmic reticulum
SERa: Smooth endoplasmic reticulum aggregation
