# Supplementary figures and images for: Does smooth endoplasmic reticulum aggregation in oocytes impact the chromosome aneuploidy of the subsequent embryos? A propensity score matching study
Source: J Ovarian Res. 2023 Mar 24;16:59. doi: 10.1186/s13048-023-01135-z (PMC10037775; doi:10.1186/s13048-023-01135-z)

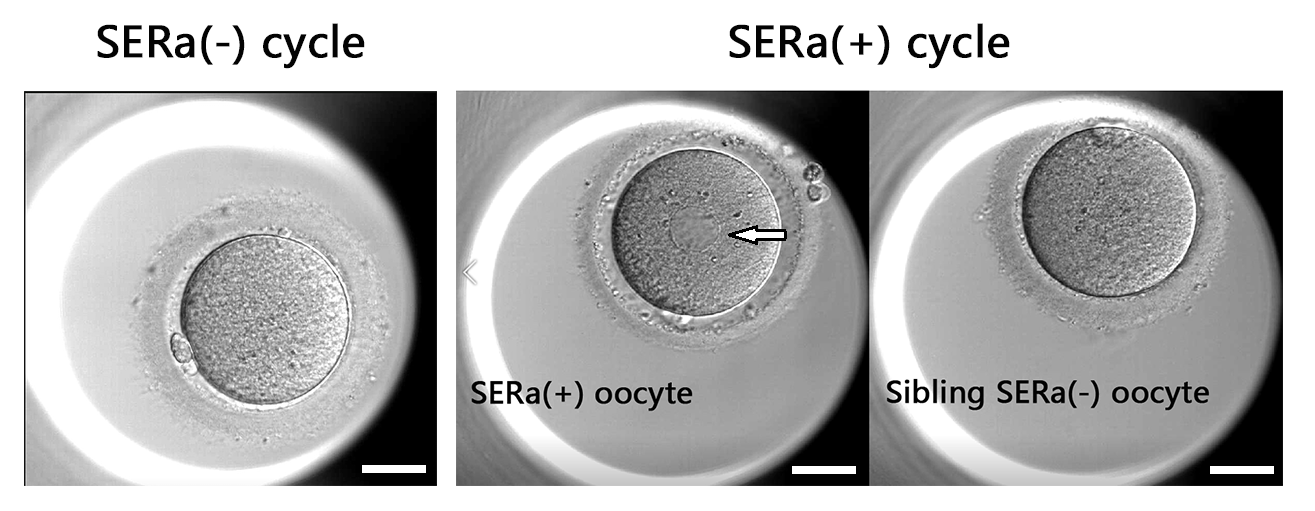

Supplement: Supplementary file 1 — Additional file 1: Figure S1. Image of SERa oocyte under time-lapse monitoring system. [file 13048_2023_1135_MOESM1_ESM.tif]
